# Guided Filter-Inspired Network for Low-Light RAW Image Enhancement

**DOI:** 10.3390/s25092637

**Published:** 2025-04-22

**Authors:** Xinyi Liu, Qian Zhao

**Affiliations:** School of Mathematics and Statistics, Xi’an Jiaotong University, Xi’an 710049, China; xinyil2525@gmail.com

**Keywords:** low-light RAW image enhancement, guided image filter, convolutional neural networks

## Abstract

Low-light RAW image enhancement (LRIE) has attracted increased attention in recent years due to the demand for practical applications. Various deep learning-based methods have been proposed for dealing with this task, among which the fusion-based ones achieve state-of-the-art performance. However, current fusion-based methods do not sufficiently explore the physical correlations between source images and thus fail to sufficiently exploit the complementary information delivered by different sources. To alleviate this issue, we propose a Guided Filter-inspired Network (GFNet) for the LRIE task. The proposed GFNet is designed to fuse sources in a guided filter (GF)-like manner, with the coefficients inferred by the network, within both the image and feature domains. Inheriting the advantages of GF, the proposed method is able to capture more intrinsic correlations between source images and thus better fuse the contextual and textual information extracted from them, facilitating better detail preservation and noise reduction for LRIE. Experiments on benchmark LRIE datasets demonstrate the superiority of the proposed method. Furthermore, the extended applications of GFNet to guided low-light image enhancement tasks indicate its broad applicability.

## 1. Introduction

Images captured in low-light environments often suffer from severe noise and color distortion, which can significantly degrade user experience and impair the performance of downstream vision tasks. To mitigate these challenges, low-light image enhancement (LIE) has garnered increasing attention in recent years [1,2,3,4,5,6]. Traditionally, LIE was mainly carried out in the sRGB domain. However, sRGB images, which are often produced by camera image signal processing (ISP) from RAW format ones, can lose valuable metadata for post-processing. As a result, direct LIE on RAW images has emerged as a new trend in recent years, following pioneering work by Chen et al. [7]. In that work, a real paired low-light RAW image dataset, See-in-the-Dark (SID), was created, and a deep learning-based pipeline was proposed to perform low-light RAW image enhancement (LRIE). This pipeline employs a deep neural network to directly map dark, noisy RAW images to bright, clean sRGB ones, simultaneously removing noise and enhancing details.

Since the introduction of this end-to-end LRIE pipeline, multiple deep learning methods [8,9,10,11,12] have been developed and demonstrated promising results. Among them, the methods that use a fusion-based approach to enhance low-light RAW images with auxiliary images, mostly self-generated from the RAW images [11,12], have achieved state-of-the-art performance. These methodologies achieve performance superiority by effectively leveraging cross-modal complementary features, particularly in preserving spatial context and high-frequency textural patterns, all of which are crucial for restoring images with high visual quality in the LRIE task. However, it is worth noting that these methods usually employ feature domain fusion, which involves directly feeding aggregated features (e.g., concatenation or average) extracted from different source images into subsequent network modules, without fully exploring the physical correlations between sources. Here, physical correlations refer to the inherent and explicit relationships between source inputs, such as consistent or complementary spatial structures, due to their derivation from the same RAW source. As a result, the complementary information from different sources may not be utilized effectively, leading to limited performance, particularly in detail preservation and noise reduction.

The spatially variant linear representation model (SVLRM) represents a significant advancement in image fusion by integrating the guided filter [13,14] with Convolutional Neural Networks (CNNs) [15]. SVLRM leverages the learnable parameters of the guided filter within the image domain to achieve effective image fusion, outperforming traditional human-designed filters. By combining the edge-preserving properties of the guided filter with the feature extraction capabilities of CNNs, SVLRM successfully addresses challenges related to detail preservation and noise reduction. However, its scope is primarily confined to the image domain, limiting its ability to fully exploit the potential of guided filtering in more abstract feature representations. To overcome this limitation, we propose a novel deep neural network, termed the Guided Filter-Inspired Network (GFNet), which extends the concept of learnable guided filtering into the feature domain. Unlike SVLRM, GFNet integrates guided filtering principles into the hierarchical feature spaces of CNNs, enabling more robust detail preservation and enhanced noise suppression through guided filtering.

The proposed GFNet primarily comprises two key modules: the Image-Guided Fusion (ImGF) module and the Feature-Guided Fusion (FeGF) module. The ImGF module enhances detail preservation by fusing two source images derived from the low-light RAW image in the image domain. While inspired by SVLRM [16], this module adheres more strictly to the original principles of the guided filter [13]. The FeGF module extends the GF-like fusion mechanism to the feature domain, enabling more effective fusion of complementary information from features extracted from different sources. This extension results in improved noise removal and overall enhancement performance.

To summarize, our main contributions are as follows:

- From the perspective of decomposing and fusing contextual and textural information, we propose a novel approach for the LRIE task. The proposed method incorporates the guided filtering mechanism into the network structure, leading to a more reasonable and effective fusion for this task.
- To better preserve details, we propose an ImGF network module to fuse input source images in the image domain, emphasizing contextual and textural information related to the low-light RAW image, respectively. In addition, we introduce an FeGF module that extends the GF-like operation to the feature domain, allowing for a more effective fusion of multi-scale features extracted from input sources, thus leading to better noise reduction. Together, these modules promote more effective fusion by better utilizing complementary information from the source images.
- By combining the ImGF and FeGF modules, along with a multi-scale feature extraction module, we construct the GFNet for the LRIE task, achieving state-of-the-art performance on the SID benchmark.

## 2. Related Work

**LIE for sRGB images.** The LIE problem was originally raised for sRGB images. The early work mainly focused on value mapping, such as histogram equalization [17,18] and gamma correction [19,20]. Later, Retinex theory [21] became more popular and was adopted as the base model in many methods [1,2,3]. Recently, deep learning-based methods for this task have achieved superior performance [4,5,22,23,24,25,26,27].

**LIE for RAW images.** As mentioned in Introduction, since RAW images can provide more useful information for post-processing, the LRIE problem has attracted increased attention in recent years. The pioneering work was conducted by Chen et al. [7], who built the SID dataset, which consists of noisy low-light RAW images captured by DSLR in extremely low-light indoor/nighttime situations and their corresponding clean normal-light ones. Additionally, the authors proposed a deep ISP pipeline with U-Net [28] to enhance noisy low-light RAW images and directly output clean normal-light sRGB ones in an end-to-end manner. On the basis of the SID dataset and the proposed pipeline, multiple deep learning methods for LRIE have been developed and have achieved distinguished performance. For example, Gu et al. [8] adopted a top–down self-guidance architecture to better exploit multi-scale features; Xu et al. [9] proposed a frequency-based decomposition and enhancement structure for sufficient noise removal; Zhu et al. [11] generated multi-exposure frames from raw sensor images in order to fuse them for higher contrast and utilized a pre-trained edge detection network for better detail preservation. Jin et al. [29] proposed a decoupled feedback framework with RAW and sRGB feature interaction to address domain ambiguity and information loss. Huang et al. [30] applied HDR reconstruction with diverse CRFs to generate multi-exposure frames from a low-light RAW image and fused them to produce high-quality sRGB images. Wang et al. [31] proposed a multi-scale feature fusion network to to fully capture and enhance global details in low-light images. Among these methods, a major clue for network design is to fuse multi-modal source images generated from the original RAW image.

**Guided Filter.** The guided filter (GF) [13,14] is a computationally efficient edge-preserving filter that leverages a guidance image to maintain structural details during smoothing, widely used in denoising and image fusion. Subsequent extensions enhance its adaptability: Li et al. [32] proposed a weighted GF with a dynamic range-aware mechanism for improved detail preservation; Zhang et al. [33] designed the Rolling Guidance Filter to iteratively separate texture and structure; Ham et al. [34] introduced non-convex potentials to enhance noise compression while preserving edges. Recent efforts integrate GF with deep learning for enhanced flexibility. Lv et al. [23] developed a Fast GF enabling end-to-end CNN joint optimization, while Dong et al. [16] proposed a CNN-driven spatially variant linear model (SVLRM) to transfer structural guidance effectively. These methods excel in enhancement, denoising, and fusion tasks. However, existing approaches remain limited by partial integration of GF principles and image-domain restrictions, underscoring the need for frameworks unifying GF’s theoretical strengths with deep learning’s representational power.

## 3. Guided Filter-Inspired Network

In this section, we first provide a brief review of the guided filter (GF) [13,14] and then introduce the detailed modules of our proposed GF-inspired network for the low-light RAW image enhancement task.

### 3.1. Preliminary and Motivation

The guided filter (GF) [13] is a versatile image processing tool designed to filter an input image P using a guidance image I, effectively fusing two source images. It has demonstrated significant effectiveness in various tasks, including image enhancement [14], image fusion [35,36], and image segmentation [37,38,39]. The core principle of GF is based on the assumption that the filtering output Q is a linear transform of the guidance image I within a local window $\omega_{k}$ centered at pixel *k*. Specifically, for each pixel *j* in the window $\omega_{k}$, the output is expressed as follows:

(1) $$Q_{j}=a_{k}I_{j}+b_{k},\;\;\;\forall j\in \omega_{k},$$

where $a_{k}$ and $b_{k}$ are linear coefficients computed as follows:

(2) $$\begin{array}{c} a_{k}=\frac{{\mathrm{Cov}(I,P)}_{k}}{\sigma_{k}^{2}+\epsilon }, \end{array}$$

(3) $$\begin{array}{c} b_{k}={\bar{P}}_{k}-a_{k}\mu_{k}. \end{array}$$

Here, $\mu_{k}$ and $\sigma_{k}^{2}$ are the mean and variance of I in the window $\omega_{k}$, ${\bar{P}}_{k}$ is the mean of P in $\omega_{k}$, and $\mathrm{Cov}{\left(I,P\right)}_{k}$ denotes the covariance between I and P in $\omega_{k}$. The final filtering output is obtained by averaging the contributions from all overlapping windows that contain pixel *j*:

(4) $$Q_{j}={\bar{a}}_{j}I_{j}+{\bar{b}}_{j},$$

where ${\bar{a}}_{j}$ and ${\bar{b}}_{j}$ are the averaged coefficients. In matrix form, the process is represented as follows:

(5) $$Q_{GF}=A_{GF}\circ I+B_{GF},$$

where $A_{GF}$ and $B_{GF}$ are matrices composed of the averaged coefficients, and ∘ denotes element-wise multiplication. This formulation highlights the efficiency and simplicity of GF in achieving edge-preserving smoothing and image fusion.

The use of GF for LRIE is motivated by its inherent ability to preserve structural and textural information while effectively suppressing noise—key attributes for the task. As detailed in the Introduction, fusion-based methods have achieved remarkable performance in LRIE by leveraging complementary information from different sources while showing limitations in fully exploring the physical correlations between sources. Since self-generated source images typically share consistent spatial properties, GF offers a natural framework for explicitly modeling the local correlations between these source images.

Recent advances in deep learning have enabled learnable guided filtering networks [16,23], which incorporate trainable parameters to enhance flexibility and achieve improved performance in tasks like image restoration and fusion. However, these methods often partially integrate the theoretical principles of GF, such as oversimplifying the learning process of $A_{GF}$ and $B_{GF}$ and ignoring the correlation between these feature maps. In addition, they are restricted to the image domain, limiting their ability to fully exploit structural guidance across modalities. To address these limitations, our work adopts a learnable GF framework while explicitly unifying its edge-preserving mechanism in both image and feature domains, enabling better detail preservation and noise compression performance.

### 3.2. Overall Network Structure and Workflow

**Figure 1:**
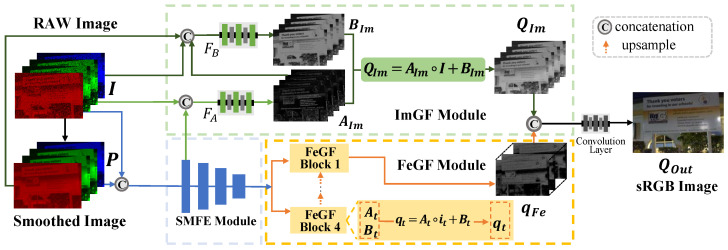
The overview of GFNet. The SMFE, ImGF, and FeGF modules are introduced in Section 3.3.1, Section 3.3.2, and Section 3.3.3, respectively. $F_{A}$ and $F_{B}$ are convolutional layers to infer $A_{Im}$ and $B_{Im}$, whose details are in Appendix A.

Our proposed GFNet comprises three key modules: a Semi-coupled Multi-scale Feature Extraction (SMFE) module for extracting multi-scale features, an Image-Guided Fusion (ImGF) module for image-domain fusion, and a Feature-Guided Fusion (FeGF) module for feature-domain fusion. In particular, the ImGF module is mainly designed to preserve image details better, while the FeGF module mainly contributes to better removing noise, as demonstrated in Section 4.2. The overall network structure is shown in Figure 1.

**Figure A1:**
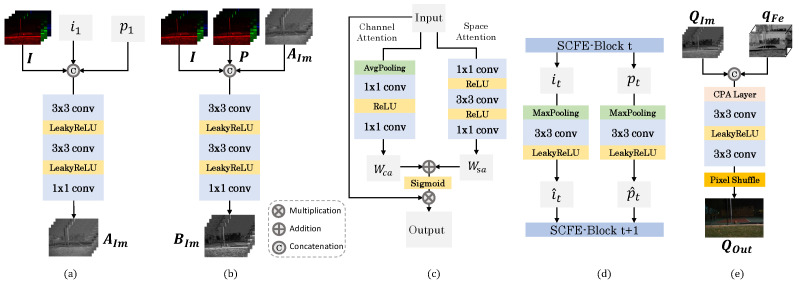
Detailed network blocks. (**a**) is the convolutional block to infer the map $A_{Im}$ in the ImGF module, as described in Equation (7). $i_{t}$ and $p_{t}$ are the abstracted features from input I and *P*; (**b**) is the convolutional block to infer the map $B_{Im}$ in the ImGF module, as described in Equation (8); (**c**) is the detailed architecture of the channel and space attention (CPA) block, as shown in Equation (12), $W_{ca}$ refers to the channel attention module, and $W_{sa}$ refers to the spatial attention module; (**d**) is the downsampling and channel expansion operations between SCFE blocks in the SMFE module, as shown in Section 3.3.1; (**e**) is the convolution layer for the combination of $Q_{Im}$ and $q_{Fe}$.

**Fusion inputs.** Building upon the property of GF, we anticipate that the guidance image will reflect more textual information, while the filtering input will provide more contextual information. Therefore, though noisy, we treat the low-light RAW image as the guidance, since it contains entire image details, as illustrated in Figure A1. As for the filtering input, we generate it by applying the bilateral filter (BF) [40] to the given RAW image, as BF provides a filtered result with reduced noise and enhanced contextual information. This choice is based on a balance between performance and simplicity, and more information and justification about it can be found in Section 4.2.

**Workflow of GFNet.** First, the low-light RAW image $I\in R^{H\times W\times 1}$ is packed to an *N*-channel tensor with a size of $\frac{H}{\sqrt{N}}\times \frac{W}{\sqrt{N}}\times N$, and we also denote it as I for convenience. Here, the number of input channels *N* is determined according to the pattern of the color filter array (CFA), e.g., N=4 for the Bayer pattern. The bilateral filtered result of I is denoted as $P\in R^{\frac{H}{\sqrt{N}}\times \frac{W}{\sqrt{N}}\times N}$. Then, P and I are input to the SMFE module to jointly extract their multi-scale features. The extracted features are then passed to the ImGF and FeGF modules for subsequent processing. In the ImGF module, the extracted features, together with the input source images, are used to infer $A_{Im}$ and $B_{Im}$ for the guided fusion result $Q_{Im}\in R^{\frac{H}{\sqrt{N}}\times \frac{W}{\sqrt{N}}\times N}$ in the image domain. In the FeGF module, the extracted multi-scale features are first fused by FeGF blocks in a GF-like way at each scale and then aggregated by upsample blocks to obtain the final feature $q_{Fe}\in R^{\frac{H}{\sqrt{N}}\times \frac{W}{\sqrt{N}}\times C}$, where *C* refers to the number of channels. Finally, $Q_{Im}$ and $q_{Fe}$ are concatenated and input to a small network with attention and convolutional layers (details are provided in Appendix A) to obtain the enhanced result $Q_{Out}\in R^{H\times W\times 3}$ with the sRGB format. The $L_{1}$ loss between $Q_{Out}$ and the ground truth $Q_{GT}$ is adopted for network training.

### 3.3. Architecture Details

In this subsection, following the workflow, we discuss the three key modules of GFNet: the SMFE module for multi-scale feature extraction, the ImGF module for image-domain fusion, and the FeGF module for feature-domain fusion. More detailed structures about GFNet are provided in Appendix A.

#### 3.3.1. SMFE Module for Multi-Scale Feature Extraction

**Figure 2:**
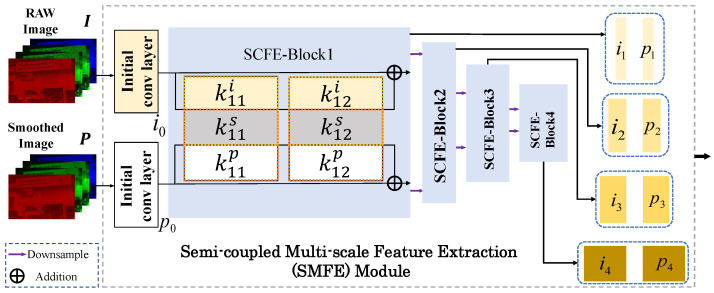
The detailed architecture of the SMFE module. $k_{1j}^{i}$ and $k_{1j}^{p}$ refer to the private convolutional kernel to extract features of I and P in the 1st SCFE block, and $k_{1j}^{s}$ refers to the shared convolutional kernel. Please refer to [41] for details of the SCFE block.

GFNet first needs to extract features from the packed RAW image I and the bilateral filtered result P. Rather than using two separate subnetworks, we introduce the semi-coupled feature extraction (SCFE) module [41] to jointly extract features from I and P, considering their high correlation, as illustrated in SCFE-Block 1 of Figure 2. With this design, the feature extractor can extract shared and private informative features effectively from input image pairs.

Also, considering the success of using multi-scale features, e.g., [28], we extend SCFE to a multi-scale version, called the SMFE module. The structure of the SMFE module is illustrated in Figure 2. Specifically, we cascade four SCFE blocks, each of which (except the first one) takes the downsampled output (obtained by max pooling and convolution operations, whose detailed architecture is presented in Appendix A) of the previous block as its input. Then, the output pairs of all SCFE blocks are retained as multi-scale features, which are denoted as ${\left\{\left(i_{t},p_{t}\right)\right\}}_{t=1}^{4}$. Each pair $(i_{t},p_{t}),t=2,3,4$ has half the spatial width and height, and twice the number of channels compared with $\left(i_{t-1},p_{t-1}\right)$.

#### 3.3.2. ImGF Module for Image-Domain Fusion

The ImGF module is designed to fuse I and P in a guided filtering way for better detail preservation. Specifically, it produces the fusion result by

(6) $$Q_{Im}=A_{Im}\circ I+B_{Im},$$

where $A_{Im}$ and $B_{Im}$ should be inferred using networks with the information of I and P. This is expected to retain the textual information of I as much as possible according to the physical model of GF discussed in Section 3.1.

As discussed before, $A_{Im}$ should characterize the intrinsic correlations between I and P, and thus it is natural to use the features extracted from them as the input of the inference network. In addition, according to Equation (2), the statistics of guidance I, i.e., variance, is adopted to normalize *A* in GF, and therefore it is reasonable to also treat I as input for inferring $A_{Im}$. Combining these two observations, we can use a small convolutional network to infer $A_{Im}$:

(7) $$A_{Im}=F_{A}\left(\left\{i_{1},p_{1},I\right\};\theta_{A}\right),$$

where $F_{A}\left(\cdot ;\theta_{A}\right)$ is the inference network parameterized by $\theta_{a}$, and $\left(i_{1},p_{1}\right)$ denotes features output by the first SCFE block in the SMFE module. Note that we only use the features with the same spatial size as I and P here, which makes the concatenation operation simpler.

According to Equation (3), the calculation for *B* in GF is based on *A*, P, and I. Therefore, we can use I and P, as well as $A_{Im}$, as the input to the inference network of $B_{Im}$:

(8) $$B_{Im}=F_{B}\left(\left\{A_{Im},P,I\right\};\theta_{B}\right),$$

where $F_{B}\left(\cdot ;\theta_{B}\right)$ is a small convolutional network parameterized by $\theta_{B}$.

With the designing mechanism discussed above, the learnable guided fusion in the image domain can be achieved by ImGF.

#### 3.3.3. FeGF Module for Feature-Domain Fusion

The FeGF module extends the GF-like operation to the feature domain, aiming at a more effective fusion of the features extracted from I and P by SMFE. Specifically, at each scale t(t=1,2,3,4), $i_{t}$ and $p_{t}$ are fused according to

(9) $$q_{t}=a_{t}\circ i_{t}+b_{t}.$$

**Figure 3:**
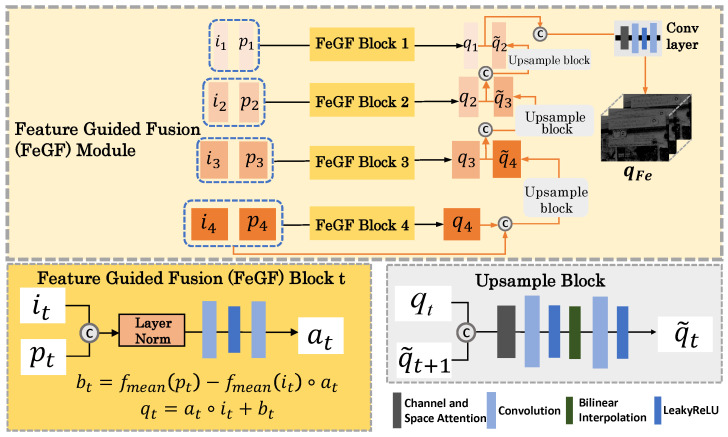
The detailed architecture of the FeGF module.

Here, $a_{t}$ is calculated by

(10) $$a_{t}=f_{a}^{t}\left(\left\{LN\left(i_{t}\right),LN\left(p_{t}\right)\right\};\theta_{a}^{t}\right),$$

where $f_{a}^{t}\left(\cdot ;\theta_{a}^{t}\right)$ is a small convolutional network parameterized by $\theta_{a}^{t}$, and LN(·) refers to layer normalization. Here, we apply layer normalization before input $\left(i_{t},p_{t}\right)$ to $f_{a}^{t}$, because its importance in low-level vision has recently been emphasized by [42]. For $b_{t}$, we directly calculate it using the following equation:

(11) $$b_{t}=f_{\mathrm{mean}}\left(p_{t}\right)-f_{\mathrm{mean}}\left(i_{t}\right)\circ a_{t},$$

where $f_{\mathrm{mean}}\left(\cdot \right)$ denotes the mean filter. Note that we do not infer $b_{t}$ using a learnable network as is carried out in the image domain. Instead, we adopt the original guided filter formulation. This design is motivated by two considerations: (1) After processing through the SMFE module, the feature pair $\left(i_{t},p_{t}\right)$ already contains sufficiently rich private and shared information jointly extracted from I and P, making further abstraction in the feature space less necessary. (2) The use of a fixed mean filter significantly reduces both the number of parameters and computational cost. Combining Equations (9)–(11), we construct one FeGF block at the $t_{th}$ feature scale, which is depicted in Figure 3.

After the scale-wise fused features, ${\left\{q_{t}\right\}}_{t=1}^{4}$, are calculated, we aggregate them with upsampling and attention-based concatenation as follows:

(12) $${\tilde{q}}_{t}=g_{t}\left(CPA\left[q_{t},{\tilde{q}}_{t+1}\right];\theta_{g}^{t}\right),\;\;t=1,2,3,4.$$

Here, CPA[·,·] refers to the concatenation operation with a channel and space attention layer, $g_{1}\left(\cdot ;\theta_{g}^{1}\right)$ is a small convolutional network, and $g_{t}\left(\cdot ;\theta_{g}^{t}\right),t=2,3,4$ denotes the upsample block illustrated in Figure 3. In addition, we define ${\tilde{q}}_{5}=\left[i_{4},p_{4}\right]$ as the supplement to upsample $q_{4}$. Then, the final output of the whole FeGF module is $q_{Fe}={\tilde{q}}_{1}$.

## 4. Experiments

In this section, we conduct experiments to verify the effectiveness of GFNet. We first evaluate the performance of GFNet on the LRIE task, with ablation studies on model ingredients. To further evaluate the generalization and transferability of our method, we also conduct tests on additional real-world scenarios.

### 4.1. Experiments on the LRIE Task

#### 4.1.1. Settings

**Datasets.** We conduct experiments on the Low-Resolution Image Enhancement (LRIE) task using the Sony and Fujifilm datasets from the SID dataset [7], which comprises extremely low-light RAW images. The Sony dataset contains images captured by the Sony α7S II Bayer sensor with a resolution of 4256×2848×1, while the Fujifilm dataset includes images captured by the Fujifilm X-Trans sensor with a resolution of 6032×4032×1. Due to their distinct Color Filter Array (CFA) patterns, the raw Bayer images in the Sony dataset are packed into 4 channels, and the raw X-Trans images in the Fujifilm dataset are packed into 9 channels before being fed into the network. We adhere to the post-processing and dataset splitting protocols described in [7,11,12] for training and testing. For the Sony dataset, we use 185 paired images for training and 50 paired images for testing. For the Fujifilm dataset, we use 193 paired images for training and 41 paired images for testing. While we report results on both datasets, we place greater emphasis on the Sony dataset, as the Bayer pattern is more prevalent in consumer devices [11,12,43]. In addition, we calculated the illumination levels for the SID dataset test set with the mean grayscale value (normalized to a 0—1 scale) of the image. It spans a broad range of 0.0002 to 0.003, reflecting diverse low-light conditions.

**Competing Methods.** We compare our approach with eight representative methods, including the original guided filter (GF) [13,14], its trainable variant Fast Guided Filter (FGF) [44], and state-of-the-art methods for the LRIE task: SID [7], EEMEFN [11], SGN [8], RED [43], LRDE [9], and DBLE [12]. For the Sony dataset, SID [7], DBLE [12], RED [43], and EEMEFN [11] are evaluated using their provided pre-trained models, while the remaining methods are retrained under our experimental settings. For the Fujifilm dataset, only EEMEFN [11] is evaluated using its provided pre-trained model, with the others are retrained under our experimental settings.

**Evaluation metrics.** Four metrics, including PSNR, SSIM, LPIPS [45], and Delta E ($▵E^{*}$) [46], are adopted for quantitative evaluation. Among these metrics, PSNR and SSIM are widely used in image restoration tasks for performance evaluation; LPIPS is a deep feature-based metric aiming at assessing the perceptual quality of an image with a reference image; $▵E^{*}$ is a metric for color distortion. In addition, the number of model parameters and FLOPs on the Sony dataset is also reported.

**Implementation details.** All experiments are implemented by PyTorch 3.7 using an NVIDIA V100 GPU. For the SID dataset, including both the Sony and Fujifilm datasets, the initial learning rate of GFNet is set to $1\times 10^{-4}$ and adjusted to $1\times 10^{-5}$ after 4000 epochs. The batch size is set to 1 and the patch size is set to 512×512. For other deep learning methods, we retrain the models following the recommendations of the original work, or directly test using the author-released models.

#### 4.1.2. Results

In this section, we present both qualitative and quantitative results of the compared methods on the benchmark datasets. To facilitate a comprehensive comparison, additional visual results are provided in Appendix B.

**Figure 4:**
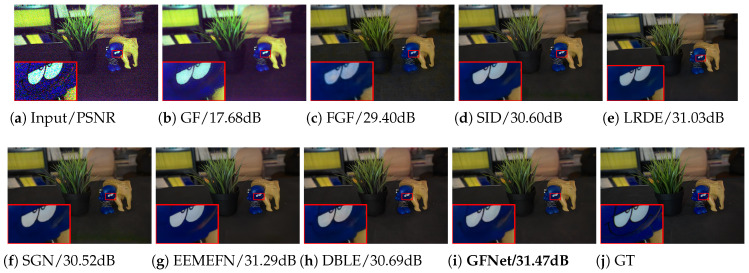
Visual results of all competing methods on the Sony dataset. The level of low-light illusion of the input image is 0.0008. The red box and magnified area in the picture mark the details that need attention. For detailed analysis, please refer to Section 4.1.2.

**Results on the Sony dataset.** The quantitative and visual results are shown in Table 1 and Figure 4, respectively. It can be seen from Table 1 that our GFNet outperforms other competing LRIE methods with respect to all the quantitative metrics. Specifically, our method outperforms all the competing methods in both PSNR and SSIM, indicating its superiority in restoring high-quality images. In addition, achieving the lowest LPIPS value indicates that our method can also produce images that are perceptually very close to the ground truth. Moreover, our GFNet also achieves the best performance with respect to the $▵E^{*}$ metric, a widely used metric to measure the color difference between two images, which evidently demonstrates its effectiveness in accurately recovering the color information against other methods. In addition to the superior average performance, the statistical indicators further demonstrate the robustness and consistency of our method. Specifically, the relatively narrow 95% confidence intervals (CIs) and small standard deviations (Stds) across all metrics indicate that GFNet produces stable results across different test samples. At the same time, in terms of model size, our method remains not too large, possessing only about one-ninth the number of parameters compared to EEMEFN, which ranks second on most evaluation metrics. In terms of computational cost and efficiency, measured by FLOPs and inference time, our method also achieves lower values than the state-of-the-art approaches DBLE [12] and EEMEFN [11], highlighting its favorable trade-off between performance and efficiency.

As for visual quality, the performance of our method is also promising. Specifically, as can be seen from Figure 4, most of the compared methods, including GF, FGF, SID, and DBLE, suffer from unexpected artifacts and uneven surfaces. While LRDE, SGN, and EEMEFN have smoother visuals, they are noticeably over-smoothed and lose important details. In comparison, our method provides a more faithful recovery with reference to the ground truth.

**Figure 5:**
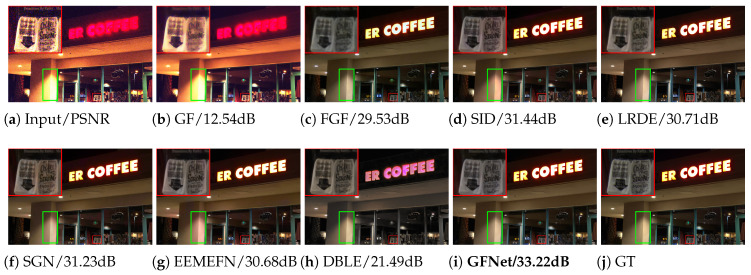
Visual results of all competing methods on the Fujifilm dataset. The level of low-light illusion of the input image is 0.0009. The red and green boxes and the magnified area in the picture mark the details that need attention. For detailed analysis, please refer to Section 4.1.2.

**Results on the Fujifilm dataset.** We present the quantitative and visual results in Table 2 and Figure 5, respectively. Compared with the Sony dataset, the Fujifilm one is more challenging due to its more complex sensor pattern. Nevertheless, our GFNet still shows its superiority. Specifically, GFNet outperforms the most competitive EEMEFN method in most adopted metrics, except for LPIPS. Considering the huge gap in the model size between the two methods, the effectiveness of the designing mechanism underlying our GFNet can be substantiated. Beyond the average values, the statistical indicators further confirm the robustness of GFNet.

Visual results in Figure 5 demonstrate that GFNet can effectively restore both textural and contextual information from the degraded input. In contrast, other competing methods either suffer from color distortion or fail to recover details with less noise, or both. For example, SID, SGN, and EEMEFN suffer from severe color bias despite producing promising structural reconstruction. Interestingly, we find that FGF and our GFNet perform visually better in color recovery, indicating that the introduction of the GF model may help to alleviate the color distortion issue.

### 4.2. Ablation Study

**Figure 6:**
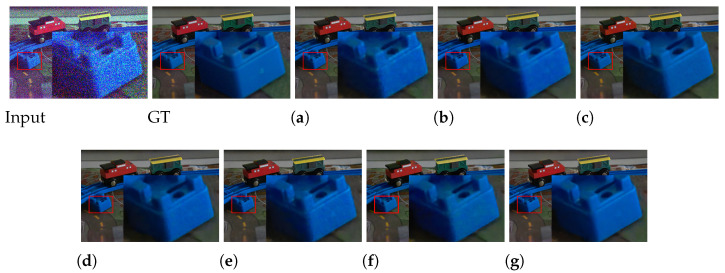
Visualization of ablation study on the Sony dataset. The order of subfigures follows Table 3.

In this subsection, we conduct experiments on the Sony dataset to verify the necessity and effectiveness of key modules in the proposed GFNet. The overall results are summarized in Table 3 and shown in Figure 6, whose full-size version is provided in Appendix B.

**Ablation on the inputs.** As discussed in Section 3.2, applying BF is a simple yet effective strategy for generating the filtering input. Therefore, we conduct experiments here to verify its validity.

We first replace the bilateral filtered image with the original RAW input, which results in somewhat of a self-guided fusion procedure. Not surprisingly, the quantitative result in Line (a) of Table 3 is not as promising as that with BF, which is also confirmed by the visual result in Figure 6a. Then, we replace the bilateral filter with a Gaussian filter, which can also suppress noise to some degree. The obtained result, though better than that without filtering, is still significantly worse compared with that of using BF, as can be observed from Line (b) of Table 3 and Figure 6b. We also replace BF with a simple UNet. At the expense of extra parameters, the results in Line (c) of Table 3 and Figure 6c are very close to that of using BF. In summary, these results demonstrate the balance between the effectiveness and simplicity of BF in our framework.

**Ablation on the fusion modules.** We conduct experiments to verify the necessity of the two key modules, ImGF and FeGF, in our GFNet. These two modules are responsible for fusion in image and feature domains, respectively, and we show that both of them play important roles.

We first replace the ImGF and FeGF modules with ordinary convolutional layers with a similar number of parameters. The results are shown in (d) and (e) of Table 3 and Figure 6. Unsurprisingly, the performance in terms of all metrics becomes worse, as can be seen from Lines (d) and (e) of Table 3. In addition, visual artifacts can be clearly observed in Figure 6d,e, especially for replacing the FeGF module.

We then experiment by replacing both ImGF and FeGF modules with convolutional layers, and the results are shown in (e) of Table 3 and Figure 6. Together with the separate ablations, two observations can be summarized. First, the performance significantly becomes worse than that of GFNet and also worse than that of replacing any of the two modules. This suggests that both modules are important to the final results. Second, ImGF and FeGF play complementary roles in the whole framework. Specifically, the visual result in Figure 6f is smoother than that in Figure 6e, indicating the denoising ability of FeGF; on the other hand, the visual result in Figure 6f has sharper edges, compared with that in Figure 6e, suggesting the detail preservation ability of ImGF.

### 4.3. Test in Real-World Scenarios

**Figure 7:**
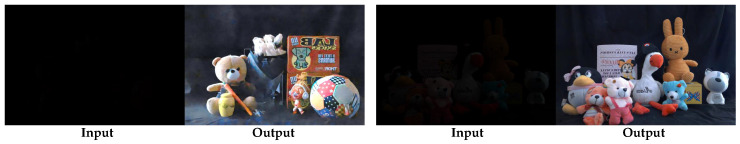
Test results of real-world scenarios.

For further comparison, we evaluate our model on real-world low-light images from [10], and the corresponding results are shown in Figure 7. The proposed method demonstrates robust enhancement performance. However, it still faces limitations under extremely challenging conditions and cross-sensor generalization challenges. For example, in the left input image, which was captured under very low illumination, the result exhibits local color biases and loss of details because of extremely heavy noise despite the overall enhancement quality.

## 5. Conclusions

In this paper, we propose a Guided Fusion Network (GFNet), inspired by guided filtering, for the low-light RAW image enhancement (LRIE) task. The proposed GFNet fuses contextual and textural information from RAW inputs through a dual-domain (image and feature) guided filtering mechanism, explicitly leveraging physical correlations such as exposure-dependent brightness complementarity, noise continuity, and spectral consistency across input sources. Extensive experiments validate the method’s superiority over state-of-the-art approaches, and extended applications in guided low-light enhancement tasks demonstrate its generalization potential for broader image fusion scenarios, such as guided depth map super-resolution [41], multi-focus image fusion [47], and 3D imaging [48]. However, as a supervised learning framework, GFNet requires paired training data with domain-specific ground truth, which may limit its adaptability to unseen sensor configurations or extremely low-light conditions beyond the training distribution. Future work could explore unsupervised or self-supervised paradigms to reduce dependence on meticulously aligned datasets while retaining physics-guided fusion principles. Additionally, current LRIE methods, including ours, are generally designed for specific sensor types and data formats (e.g., Bayer or xTrans). There is potential for further research to develop more generalizable approaches, or even an all-in-one method, capable of handling a wider range of sensor types and formats. Moreover, for practical deployment, especially on devices with limited computational resources, model efficiency becomes a crucial consideration. In such resource-constrained scenarios, the computational burden of GFNet can be alleviated through model compression techniques such as knowledge distillation or by reducing channel dimensions, enabling a better balance between performance and efficiency.

## Figures and Tables

**Table 1:** Quantitative results on Sony dataset with statistical metrics (95% confidence intervals). The best and second-best results are highlighted in bold and underlined, respectively.

Method	PSNR ↑		SSIM ↑		LPIPS ↓		$▵E^{*}↓$		Param. (M)	FLOPs (G)	Time (s)
	Mean ± CI	Std	Mean ± CI	Std	Mean ± CI	Std	Mean ± CI	Std	↓	↓	↓
GF	16.03 ± 1.51	0.20	0.519 ± 0.05	0.20	0.410 ± 0.03	0.12	18.76 ± 2.50	8.06	-	-	0.001
FGF	28.86 ± 1.04	3.65	0.772 ± 0.04	0.13	0.311 ± 0.04	0.15	5.04 ± 0.49	1.73	0.035	2.584	0.003
SID	29.39 ± 1.95	5.12	0.794 ± 0.06	0.16	0.240 ± 0.06	0.16	5.25 ± 0.88	2.32	7.761	54.98	0.009
LRDE	29.54 ± 1.03	3.63	0.798 ± 0.04	0.13	0.253 ± 0.04	0.13	4.72 ± 0.49	1.72	8.621	138.2	0.014
SGN	29.46 ± 1.19	4.17	0.800 ± 0.04	0.13	0.249 ± 0.04	0.14	4.94 ± 0.56	1.97	4.113	57.59	0.010
EEMEFN	30.07 ± 1.25	4.38	0.801 ± 0.04	0.13	0.247 ± 0.04	0.14	4.66 ± 0.60	2.10	85.24	702.1	0.021
RED	28.66 ± 2.00	7.04	0.790 ± 0.10	0.35	0.312 ± 0.11	0.39	20.17 ± 2.20	5.78	0.785	5.173	0.005
DBLE	29.70 ± 1.40	4.93	0.796 ± 0.05	0.18	0.247 ± 0.07	0.25	4.76 ± 1.00	2.52	15.01	100.5	0.015
GFNet (Ours)	**30.48 ± 1.24**	4.06	**0.805 ± 0.04**	0.13	**0.245 ± 0.04**	0.14	**4.50 ± 0.49**	1.72	9.742	72.47	0.012

**Table 2:** Quantitative results on Fujifilm dataset with statistical metrics (95% confidence intervals). The best and second-best results are highlighted in bold and underlined, respectively.

Method	PSNR ↑		SSIM ↑		LPIPS ↓		$▵E^{*}↓$	
	Mean ± CI	Std	Mean ± CI	Std	Mean ± CI	Std	Mean ± CI	Std
GF	11.77 ± 0.81	2.56	0.467 ± 0.05	0.16	0.515 ± 0.04	0.11	27.07 ± 1.78	5.62
FGF	27.40 ± 1.28	4.05	0.713 ± 0.07	0.21	0.418 ± 0.06	0.17	6.51 ± 1.03	3.26
SID	28.24 ± 1.30	4.13	0.745 ± 0.07	0.22	0.333 ± 0.06	0.20	6.30 ± 0.97	3.07
LRDE	26.64 ± 2.00	5.04	0.713 ± 0.11	0.29	0.380 ± 0.10	0.25	6.92 ± 1.80	3.23
SGN	28.03 ± 1.33	4.21	0.742 ± 0.07	0.22	0.343 ± 0.06	0.20	6.36 ± 1.00	3.16
EEMEFN	28.66 ± 1.23	4.49	0.743 ± 0.07	0.22	**0.315 ± 0.06**	0.18	6.20 ± 0.92	3.92
RED	16.15 ± 2.80	4.85	0.500 ± 0.20	0.70	0.595 ± 0.18	0.63	16.32 ± 1.50	5.83
DBLE	24.05 ± 2.50	4.79	0.669 ± 0.15	0.53	0.372 ± 0.12	0.42	8.38 ± 2.20	3.74
GFNet (Ours)	**29.39 ± 1.37**	4.35	**0.747 ± 0.07**	0.22	0.352 ± 0.06	0.20	**5.69 ± 0.99**	3.15

**Table 3:** Quantitative results of ablation study on the Sony dataset.

	PSNR ↑	SSIM ↑	Param. (M) ↓
(a) w/o BF	29.96	0.792	9.742
(b) BF ⇒ Gauss	30.04	0.796	9.742
(c) BF ⇒ UNet	30.62	0.804	11.07
(d) ImGF ⇒ Conv	30.13	0.800	9.727
(e) FeGF ⇒ Conv	29.99	0.797	9.742
(f) ImGF&FeGF ⇒ Conv	29.49	0.792	9.727
(g) GFNet (Ours)	30.61	0.808	9.742

**Figure A2:**
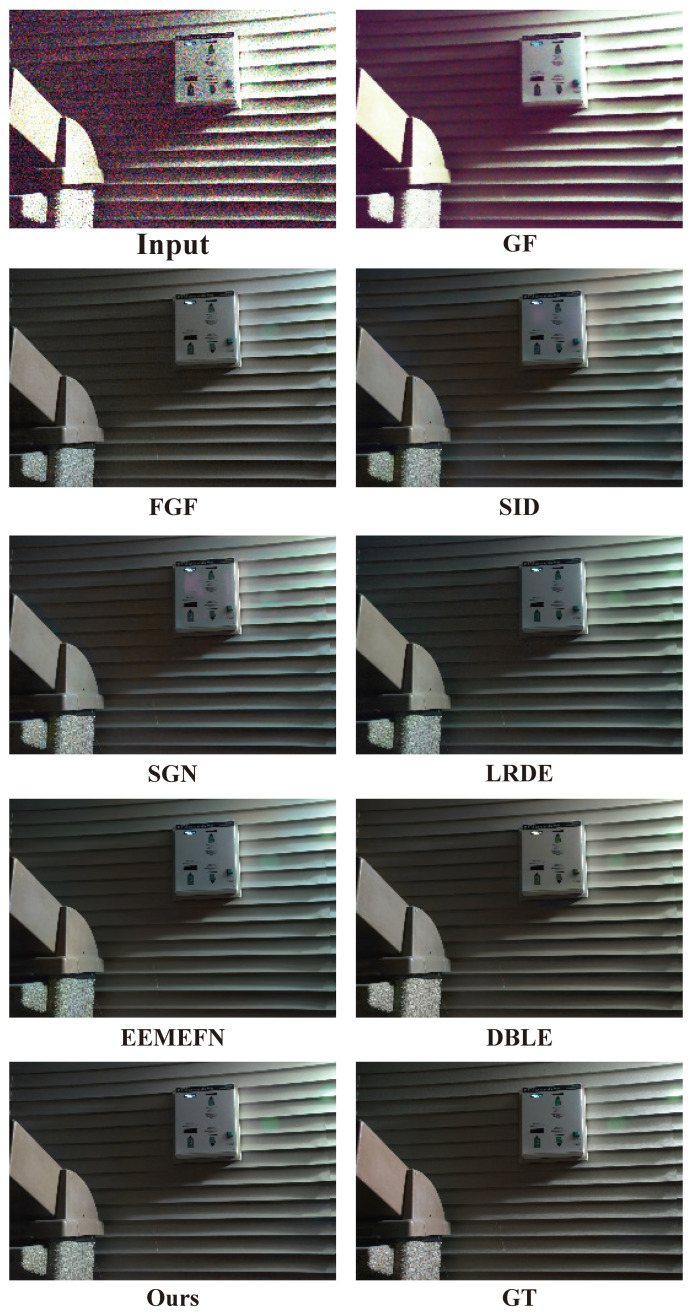
Visual comparison of SOTA methods on the Sony Dataset.

**Figure A3:**
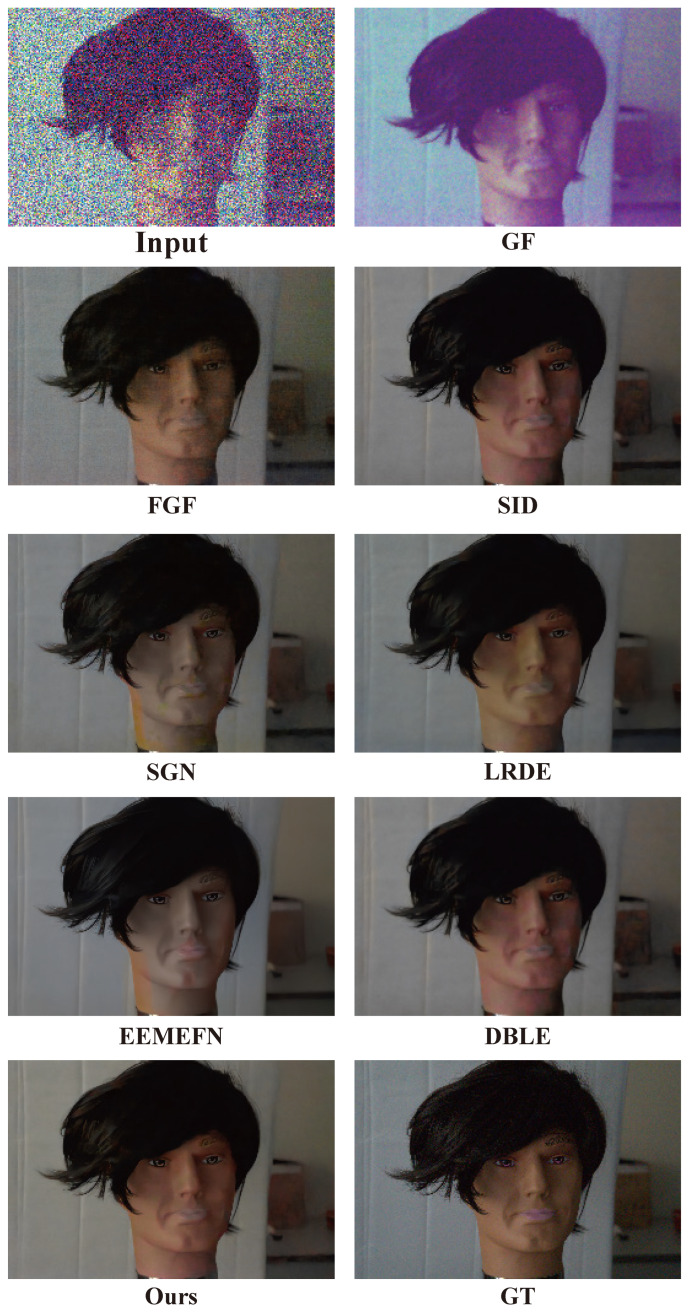
Visual comparison of SOTA methods on the Sony dataset.

**Figure A4:**
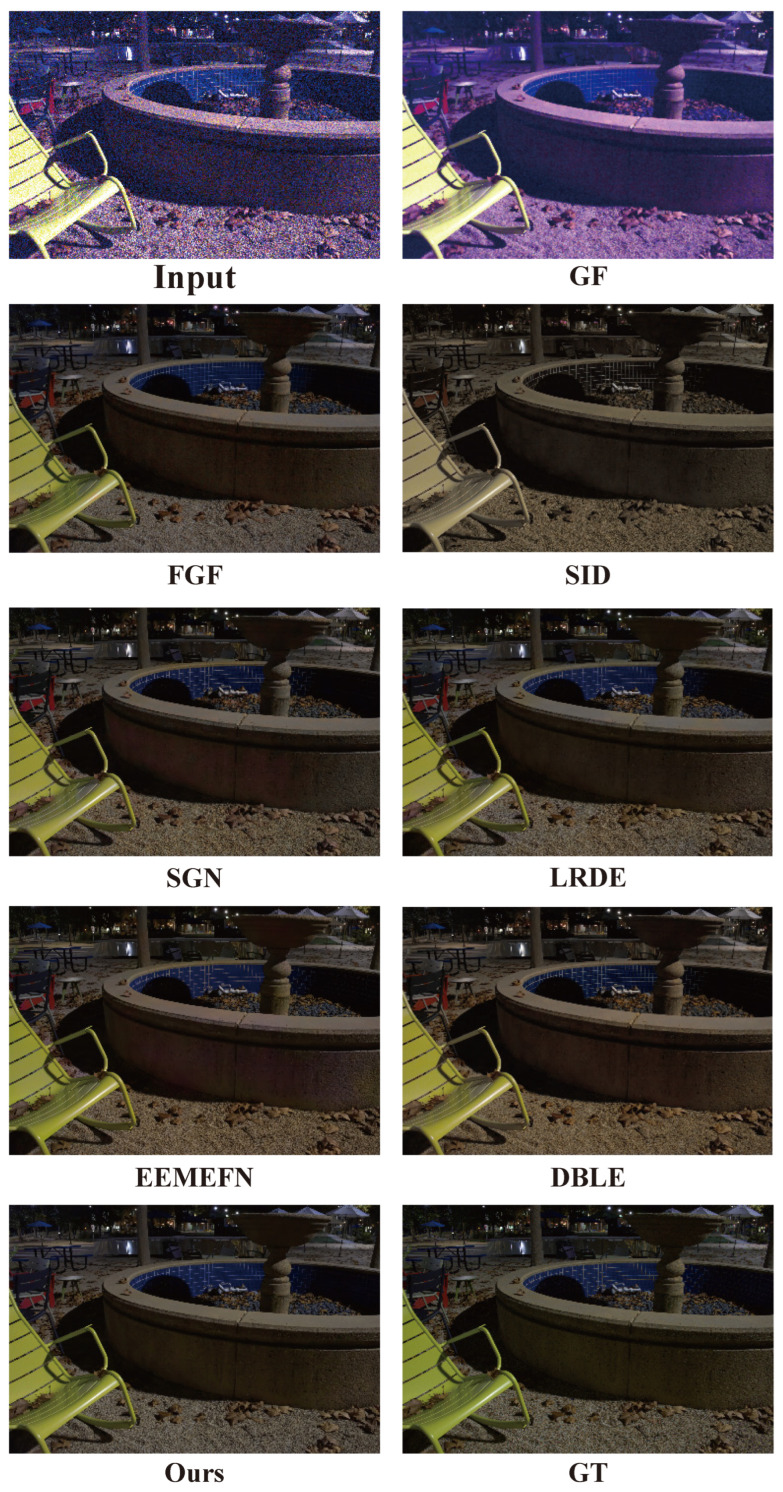
Visual comparison of SOTA methods on the Fujifilm dataset.

**Figure A5:**
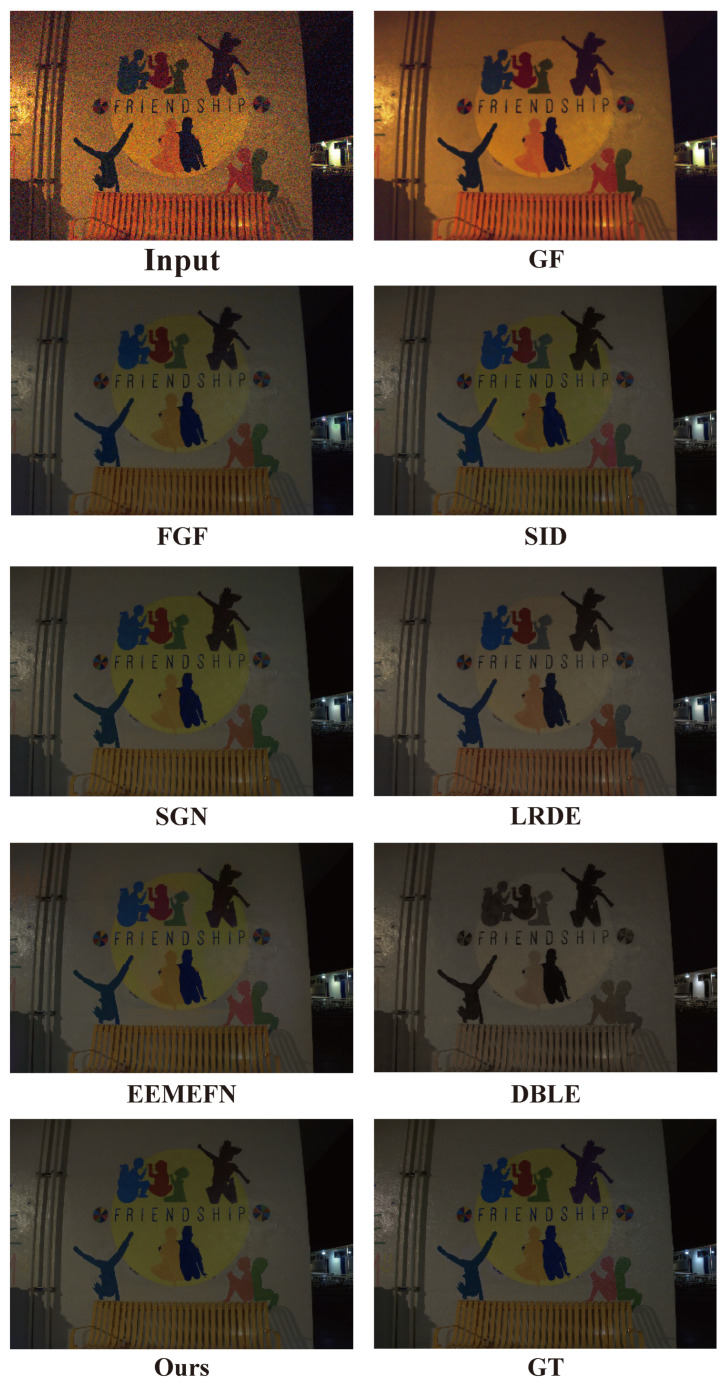
Visual comparison of SOTA methods on the Fujifilm dataset.

## Data Availability

The data presented in this study are publicly available data (sources stated in the citations). Please contact the corresponding author regarding data availability.

## Appendix A. Guided Fusion Network

In this section, we will present additional details of our network in Figure A1.

## Appendix B. Additional Evaluation Results

Complementary visualization results of Sony and Fujifilm datasets are shown in Figure A2 and Figure A3 and Figure A4 and Figure A5, respectively.
